# Analysis of the Outer Membrane Proteome and Secretome of *Bacteroides fragilis* Reveals a Multiplicity of Secretion Mechanisms

**DOI:** 10.1371/journal.pone.0117732

**Published:** 2015-02-06

**Authors:** Marlena M. Wilson, D. Eric Anderson, Harris D. Bernstein

**Affiliations:** 1 Genetics and Biochemistry Branch, National Institute of Diabetes and Digestive and Kidney Diseases, National Institutes of Health, Bethesda, MD, United States of America; 2 Advanced Mass Spectrometry Facility, National Institute of Diabetes and Digestive and Kidney Diseases, National Institutes of Health, Bethesda, MD, United States of America

## Abstract

*Bacteroides fragilis* is a widely distributed member of the human gut microbiome and an opportunistic pathogen. Cell surface molecules produced by this organism likely play important roles in colonization, communication with other microbes, and pathogenicity, but the protein composition of the outer membrane (OM) and the mechanisms used to transport polypeptides into the extracellular space are poorly characterized. Here we used LC-MS/MS to analyze the OM proteome and secretome of *B. fragilis* NCTC 9343 grown under laboratory conditions. Of the 229 OM proteins that we identified, 108 are predicted to be lipoproteins, and 61 are predicted to be TonB-dependent transporters. Based on their proximity to genes encoding TonB-dependent transporters, many of the lipoprotein genes likely encode proteins involved in nutrient or small molecule uptake. Interestingly, protease accessibility and biotinylation experiments indicated that an unusually large fraction of the lipoproteins are cell-surface exposed. We also identified three proteins that are members of a novel family of autotransporters, multiple potential type I protein secretion systems, and proteins that appear to be components of a type VI secretion apparatus. The secretome consisted of lipoproteins and other proteins that might be substrates of the putative type I or type VI secretion systems. Our proteomic studies show that *B. fragilis* differs considerably from well-studied Gram-negative bacteria such as *Escherichia coli* in both the spectrum of OM proteins that it produces and the range of secretion strategies that it utilizes.

## INTRODUCTION

While advances in metagenomics have led to a greater appreciation of the diversity of organisms in the intestine and the role of the gut microbiota in human health and disease, the biology of individual gut microorganisms has not been well characterized. *Bacteroides fragilis* is a widely distributed member of the human gut microbiome that is of particular interest. This organism is involved in a variety of activities that influence human health including polysaccharide digestion, gut development and maturation, and modulation of the immune system [1,2]. *B. fragilis* provides protection from diseases such as inflammatory bowel disease and multiple sclerosis [3–5]. In addition to its role as a commensal microorganism, *B. fragilis* is an opportunistic pathogen associated with the majority of anaerobic infections, most commonly intra-abdominal sepsis [6]. Specific “toxigenic” strains of *B. fragilis* have also been associated with an increased risk for colon cancer [7].

Cell surface and secreted molecules produced by *B. fragilis* likely play important roles in colonization and persistence, communication with other microbes, and pathogenicity, but the components of the outer membrane (OM) proteome and secretome under different conditions and the mechanisms used to transport polypeptides into the extracellular space are poorly characterized. Our current understanding of the composition of the OM is derived primarily from *in silico* studies of the genome sequence and a recent analysis of the composition of OM vesicles [2,8–13]. The *B. fragilis* genome is predicted to encode almost 100 TonB-dependent transporters (TBDTs) that are presumably involved in the uptake of nutrients and small molecules including polysaccharides, iron and cobalamin [14]. Given that *E. coli* is thought to produce only nine TonB-dependent transporters [10], the large number of these proteins is striking. Curiously, nearly 70 of the predicted TBDTs, including Omp200 [15], are homologous to SusC, a well-studied starch transporter produced by *B. thetaiotaomicron* [2]. Besides the TBDTs, *B. fragilis* appears to produce a variety of other putative integral OM proteins, but the function of many of these proteins cannot be predicted from their sequences. Based on the presence of a characteristic lipobox motif [16], the *B. fragilis* genome also appears to encode about twice as many lipoproteins as *E. coli* K-12 (177, or 4.2% of the total proteome vs. 86, or 2.0% of the total proteome; see ref. [17]). The rules that govern the localization of lipoproteins in *B. fragilis* have not been elucidated, however, so the fraction of these proteins that are released from the inner membrane (IM) and targeted to the OM is unclear. Twenty of the predicted lipoproteins are homologous to SusD, a protein that is required for starch binding. Furthermore, like the genes that encode SusD and three other lipoproteins that facilitate starch utilization (SusE, SusF and SusG), a large proportion of the genes that encode lipoproteins are adjacent to genes that encode TBDTs and likely promote the internalization or metabolism of unidentified carbohydrates or small molecules. Recently, a TBDT and a SusD homolog were shown to be components of Sus-like systems that mediate uptake and metabolism of xyloglucans and sialic acid in *Bacteroides* species [18].

Only a few OM proteins other than SusC-G have been examined experimentally. These proteins include a lipoprotein that binds plasminogen [19], a group of proteins involved in autoaggregation (Aap proteins) [20] and several cell surface glycoproteins [21]. *B. fragilis* has also been shown to produce homologs of the *E. coli* OmpA protein, but their function is unknown [22]. Binding assays have been used to show that *B. fragilis* adheres to mammalian cells and host factors including laminin-1, fibronectin, fibrinogen and mucin [23–28]. The identity of the proteins that mediate these binding activities remains unclear, however, in part because many of the studies were performed before the genome sequence was available. Nevertheless, there is evidence for the involvement of lectin-like proteins, a surface glycoprotein and a TBDT [25,26,29]. A high throughput transposon mutagenesis screen has also suggested that specific OM proteins promote fitness or survival of *B. thetaiotaomicron* in the mouse intestine and/or in laboratory media [30].

While limited information about *B. fragilis* OM proteins has emerged from bioinfomatic and experimental studies, the nature and identity of proteins that are secreted into the extracellular space is completely unknown. Available evidence indicates that *B. fragilis* secretes bacteriocins [31,32], but polypeptides that mediate antimicrobial activities have not been purified. Furthermore, it is possible that *B. fragilis* secretes proteases, glycosidases and hemolysins that facilitate nutrient and iron uptake as well as proteins that modulate the activity of host cells. Unlike OM proteins, however, secreted proteins are often difficult to predict using sequence analysis. Secreted proteins often lack N-terminal signal peptides because they are targeted to specialized secretion systems that bypass the Sec pathway. The targeting signals are often poorly conserved and therefore difficult to detect. Even when secreted proteins contain N-terminal signal peptides and reach the periplasm via the Sec pathway, they are often earmarked for secretion by non-linear or structure based targeting signals. To complicate matters, many of the protein secretion systems that have been characterized in Proteobacteria do not appear to exist in the Bacteroidetes. The *B. fragilis* genome encodes homologs of TolC, an OM protein that is used in conjunction with multidrug resistant pumps (MDRs) to export antimicrobial compounds and with ABC transporters to secrete proteins via the type I secretion system, but the expression of these genes has not been characterized. Interestingly, the *B. fragilis* genome also encodes a type VI secretion system that is highly divergent from the canonical system encoded by Proteobacteria. This secretion system has been shown to be produced during colonization of germ-free mice and upon exposure to *B. thetaiotaomicron* [33].

In order to obtain insight into the spectrum of proteins localized to or transported across the cell surface of *B. fragilis*, we analyzed the OM proteome and secretome of a common reference strain grown under laboratory conditions using mass spectrometry and methods that assess cell surface exposure. We found that a large number of TBDT and lipoproteins are produced constitutively in both rich medium and minimal media containing different carbon sources. The results suggest that the expression of genes that encode nutrient transporters is less highly regulated by substrate concentration than the expression of their *E. coli* counterparts. Interestingly, the results also indicated that a significant percentage of the lipoproteins are exposed on the cell surface. While the cell surface exposure of a few lipoproteins has been described in organisms such as *Borrelia burgdorferi, Legionella pneumophila* and *Neisseria gonorrhoeae* [34–36], extensive transport of lipoproteins across the OM has not been reported. Furthermore, we found that *B. fragilis* produces three proteins that belong to a novel family of autotransporters, several TolC-like proteins that are encoded adjacent to genes that likely encode components of type I secretion systems, and components of the type VI secretion apparatus. While the substrates for these putative type I and type VI secretion systems are unknown, they may include a subset of the diverse group of uncharacterized proteins that are secreted into the culture medium. Taken together, our results strongly suggest that *B. fragilis* uses both well-established and novel mechanisms to translocate proteins across the OM.

## MATERIALS AND METHODS

### Bacterial growth conditions

*B. fragilis* NCTC 9343 was grown at 37°C in prereduced TYG (tryptone/yeast extract/glucose) broth or pre-reduced defined minimal medium (carbohydrate source added at a final concentration of 0.5%) [37] to exponential phase. Cells were collected by centrifugation at 4,000 x g for 20 min at 4°C.

### Isolation of OM and culture medium fractions

OM fractions were prepared using a modified version of the method employed by Kotarski and Salyers [38]. Cells were washed with 10 mM HEPES, pH 7.3 and collected by centrifugation (4,000 x g, 15 min, 4°C). Washed cells were resuspended in 10 mM HEPES, pH 7.3 or PBS (phosphate buffered saline)/10% sucrose and broken by sonication using a Misonix 3000 sonicator, (level 5, 30 sec on/30 sec off/30 sec on). Unbroken cells and debris were removed by centrifugation (17,000 x g, 15 min, 4°C). The crude cell extract was applied to a step gradient containing 37% and 70% sucrose layers and centrifuged in a Beckman SW 41 Ti rotor at 34,000 rpm for 1 h at 4°C. The cloudy material between the sucrose layers was collected and membranes were pelleted by ultracentrifugation (100,000 x g, 45 min, 4°C). Mass spectrometry analysis showed that the supernatant was more highly enriched in predicted cytoplasmic proteins than the final OM fraction. The pellet was resuspended in 10 mM HEPES, pH 7.3/0.3% Sarkosyl/0.05 mM phenylmethylsulfonyl fluoride (PMSF) and incubated on a rocker at room temperature for 1–2 h to solubilize IM proteins. The OM fraction was collected by ultracentrifugation (100,000 x g, 45 min, 4°C) and resuspended in 10 mM HEPES, pH 7.3 or PBS. We found that the use of a sucrose gradient greatly reduced the contamination of the OM fraction with predicted cytoplasmic proteins. To obtain culture supernatant (secreted protein) fractions, cells were removed by centrifuging culture samples twice at 4,000 x g for 15 min at 4°C. The resulting supernatants were then centrifuged at 100,000 x g for 30 min at 4°C to remove OM vesicles and insoluble material.

### Protease treatment

To analyze proteins exposed on the cell surface, cells from a 500 ml culture were washed with PBS, resuspended in 40 ml PBS containing 2 mg/ml proteinase K (PK) and incubated for 3 h at 37°C. The PK digestion was then stopped by adding 2.5 mM PMSF and incubating cells on ice for 10 min. For the PK treatment of purified membranes, cells were lysed and a total membrane fraction was obtained by step gradient fractionation and ultracentrifugation as described above. The membrane pellet was resuspended in PBS containing 0.2 mg/mL PK and incubated on ice for 30 min. The protease digestion was then stopped by adding 2.5 mM PMSF and incubating the membranes on ice for 10 min. The membranes were washed three times with cold PBS containing 2.5 mM PMSF and pelleted after each wash step by centrifugation at 100,000 x g for 45 min at 4°C. IM proteins were then solubilized and an OM fraction was obtained as described above.

### Biotinylation of cell surface proteins

Cells were washed 3 times with cold PBS, resuspended in PBS containing 1 mg/ml Sulfo-NHS-SS-biotin (Thermo Scientific), and incubated at room temperature for 30 min. Cells were collected by centrifugation (4,000 x g, 20 min, 4°C), washed three times with PBS containing 500 mM glycine, resusupended in PBS, and lysed by sonication as described above. Unbroken cells and debris were then removed by centrifuging the cell lysate at 17,000 x g for 20 min at 4°C. To obtain cell membranes, the resulting supernatant was centrifuged at 100,000 x g for 50 min at 4°C. The membrane pellet was resuspended in PBS containing 5% Elugent and 15% NeutrAvidin agarose (Thermo Scientific) and rotated for 2 h at 4°C. Biotinylated proteins were then eluted in Thermo sample buffer according to the manufacturer’s instructions.

### ^3^H-palmitic acid labeling and fluorography

Cultures grown to early exponential phase in TYG broth were incubated with 100 μCi ^3^H-palmitic acid (PerkinElmer) for 2 h at 37°C. Cells were collected by centrifugation (4,000 x g, 20 min, 4°C), washed twice with PBS, and divided into two aliquots. One aliquot was treated with 2 mg/ml PK as described above. After PK treatment, both aliquots were lysed by sonication and centrifuged (18,000 x g, 20 min, 4°C) to remove unbroken cells and debris. The cleared lysates were then centrifuged at 100,000 x g for 45 min at 4°C, and the resulting membrane pellets were resuspended in PBS. Proteins were resolved by SDS-PAGE on 8–16% Tris-glycine minigels (Life Technologies). Gels were fixed, treated with Enlightening enhancer (Kodak) and dried. Radiolabeled proteins were then detected using Kodak Biomax XAR film.

### Protein identification by mass spectrometry

For in-gel identification by mass spectrometry, proteins were first concentrated by acetone precipitation. The proteins were then resuspended in Laemmli sample buffer (without a reducing agent) and incubated with 5.3 mM DTT at 60°C for 10 min followed by 26.3 mM iodoacetamide at room temperature for 30 min. Alkylated proteins were heated to 99°C for 5 min and separated by SDS-PAGE on 10% Tris-glycine gels (OM proteins) or 15% Tris-glycine gels (secreted proteins). Gels were stained with Colloidal Blue (Invitrogen) according to the manufacturer’s instructions.

Details of the mass spectrometry methods are provided in Supporting Materials and Methods (S1 Text). In brief, mixtures of soluble proteins and proteins contained in gel slices were digested into peptide fragments using standard procedures [39–42]. Data was collected using a Waters NanoAcquity UHPLC system interfaced with a Thermo Elite mass spectrometer. Very long gradients were used in most experiments, but short gradients were used in gel-based experiments. Stable isotope dimethyl labeling was used to compare levels of individual proteins in PK-treated and untreated samples, cells grown in media containing different carbon sources, and the culture medium versus the whole cell extract. Data obtained from mixtures of soluble proteins was analyzed using MaxQuant with LFQ type comparisons as well as parent style relative quantitative measurements [43,44]. Biotinylation was assessed by identifying peptides that contained CAM-thiopropanoyl modified lysine residues. Gel-based samples were analyzed using Mascot [45].

### Bioinformatic analysis

Proteins were categorized based on results of SignalP v.4.0 using default settings for Gram negative bacteria [46], LipoP v.1.0 [47], SecretomeP v.2.0 [48], TMHMM v.2.0 [49], and PRED-TMBB (Viterbi method) [50]. Subcellular localization was determined using CELLO v.2.5 (set for Gram negative bacteria) [51]. BLAST searches were run against the non-redundant (nr) database at http://blast.ncbi.nlm.nih.gov/ using default settings. Protein sequence alignments were carried out using ClustalW2 (BLOSUM protein weight matrix, gap open 10 and gap extension 0.1) [52]. PSIPRED v3.3 was used for secondary structure prediction [53].

## RESULTS

### Identification and classification of *B. fragilis* OM proteins

We used shotgun proteomics to compile a list of OM proteins that are produced by the non-toxigenic *B. fragilis* strain NCTC 9343 grown in rich (TYG) medium. Four or more unique peptides were identified from 326 proteins that were present in purified OM fractions (S1 Table). A smaller number of peptides were identified from another group of proteins, but most of these proteins lack signal peptides (S2 Table). Because this group is highly enriched in proteins that are unlikely to be OM proteins, we excluded it from our dataset. We next used SignalP [46], LipoP [47], SecretomeP [48], TMHMM [49] PRED-TMBB [50] and CELLO [51] to predict the structure and localization of the initial set of potential OM proteins. We also searched for the presence of conserved architectural features that are annotated in the NCBI Conserved Domain Database and similarities to characterized proteins using BLAST. Based on our analysis, we excluded an additional 97 proteins that are not predicted to contain a signal peptide from the OM protein dataset. Sixty-six of these proteins are classified as cytoplasmic proteins, 28 are predicted to be inner membrane (IM) proteins, and three are predicted to be proteins that are secreted by a non-classical mechanism (S1 Table).

**Fig 1:**
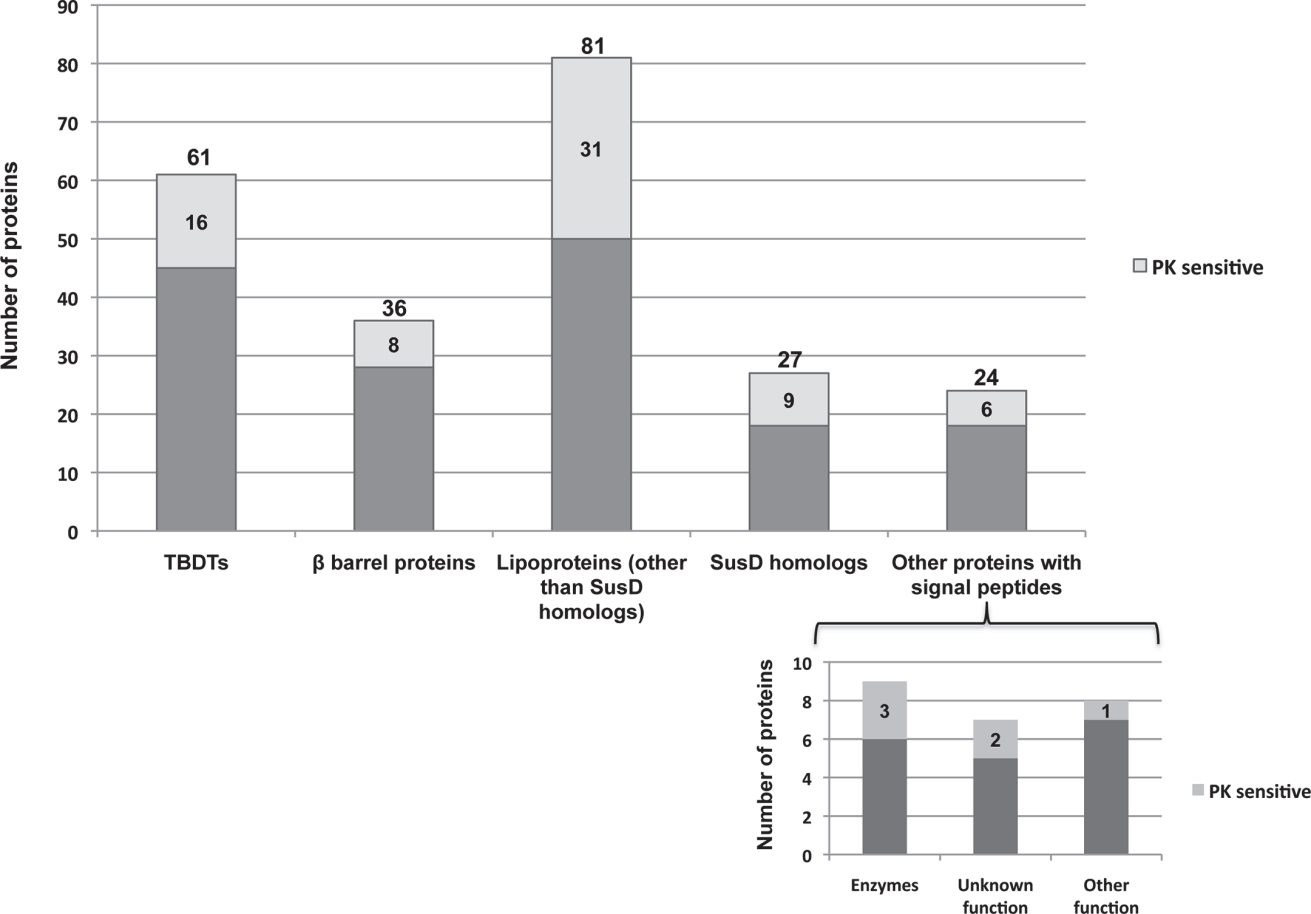
Classification of proteins in the OM proteome. Proteins in the OM fraction of cells that were treated with PK or left untreated were identified by LC-MS/MS and classified based on an analysis of their sequences. Differences in the protein composition of treated and untreated samples were assessed using label-free quantitative proteomics. A protein was considered to be PK-sensitive if protease treatment resulted in a >5-fold reduction in signal.

**Fig 2:**
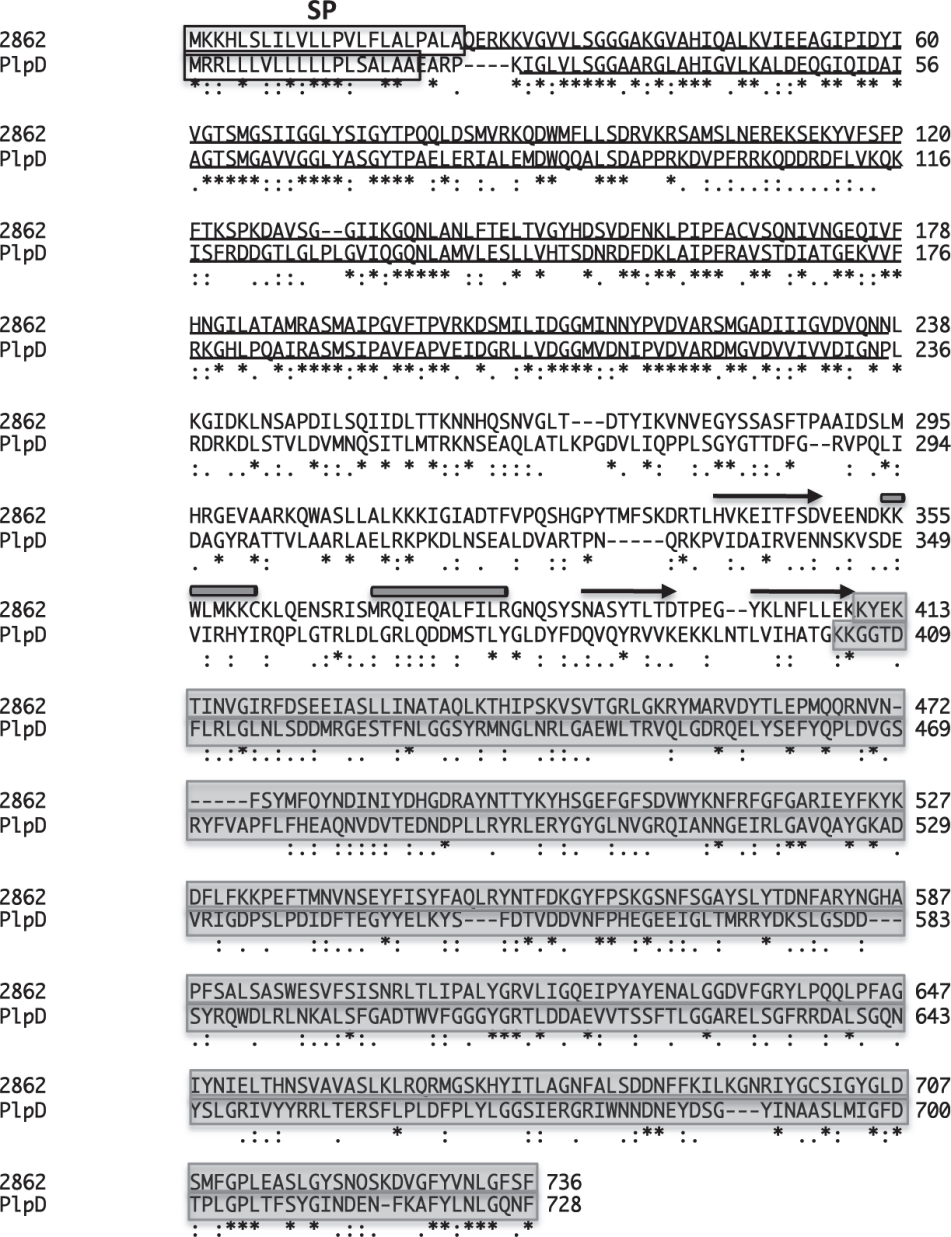
Protein BF9343_2862 shares sequence and structural homology with a prototypical member of a novel family of autotransporter-like proteins. The sequences of BF9343_2862 and *P. aeruginosa* PlpD were aligned using ClustalW2. The predicted signal peptide (SP) (boxed), patatin domain (underlined) and β barrel domain (shaded) of each protein are shown. The characteristic αββαα structure of the predicted POTRA domain (which is located N-terminal to the β barrel domain) is also highlighted.

Our bioinformatics analysis strongly suggested that the vast majority of the remaining 229 proteins are bona fide OM proteins. A total of 97 proteins are predicted to contain a β barrel domain, a membrane-spanning segment that is a hallmark of bacterial integral OM proteins [54] (Fig. 1). Sixty-one of the proteins within this group are predicted to be TBDTs. The other 36 proteins include putative porins, OmpA homologs, efflux pumps and BamA, an essential component of the β barrel assembly machinery [55]. Intriguingly, one of the predicted β barrel-containing proteins (BF9343_2862) is highly homologous to the Pseudomonas aeruginosa PlpD protein, the archetypical member of a novel (type Vd) secretion system. Two closely related proteins, BF9343_2409, and BF9343_3500, were also identified, but were represented by fewer than four peptides. PlpD consists of an N-terminal secreted domain (patatin-like domain) that is linked to a putative 16-stranded β barrel by a POTRA domain [56]. This is a unique hybrid architecture in which the N terminus is reminiscent of the extracellular domains of autotransporters, while the C terminus is reminiscent of two-partner secretion pathway transporters. The N terminus of BF9343_2862 is closely related to the patatin-like domain of PlpD (Fig. 2). Furthermore, secondary structure analysis using PSIPRED [53] revealed the presence of a POTRA domain (a three-stranded β sheet and two α helices) in BF9343_2862 that is not obvious from the sequence alignment. In addition, 108 of the 229 putative OM proteins that we identified are predicted to be lipoproteins, 27 of which are homologous to the SusD protein of *B. thetaiotaomicron*. The remaining 24 proteins include proteins that have been annotated as enzymes and proteins of unknown function [12]. This group may contain proteins that are peripherally associated with the OM, bound to OM proteins, or embedded in the OM by a novel mechanism that does not involve the formation of a β barrel structure.

**Fig 3:**
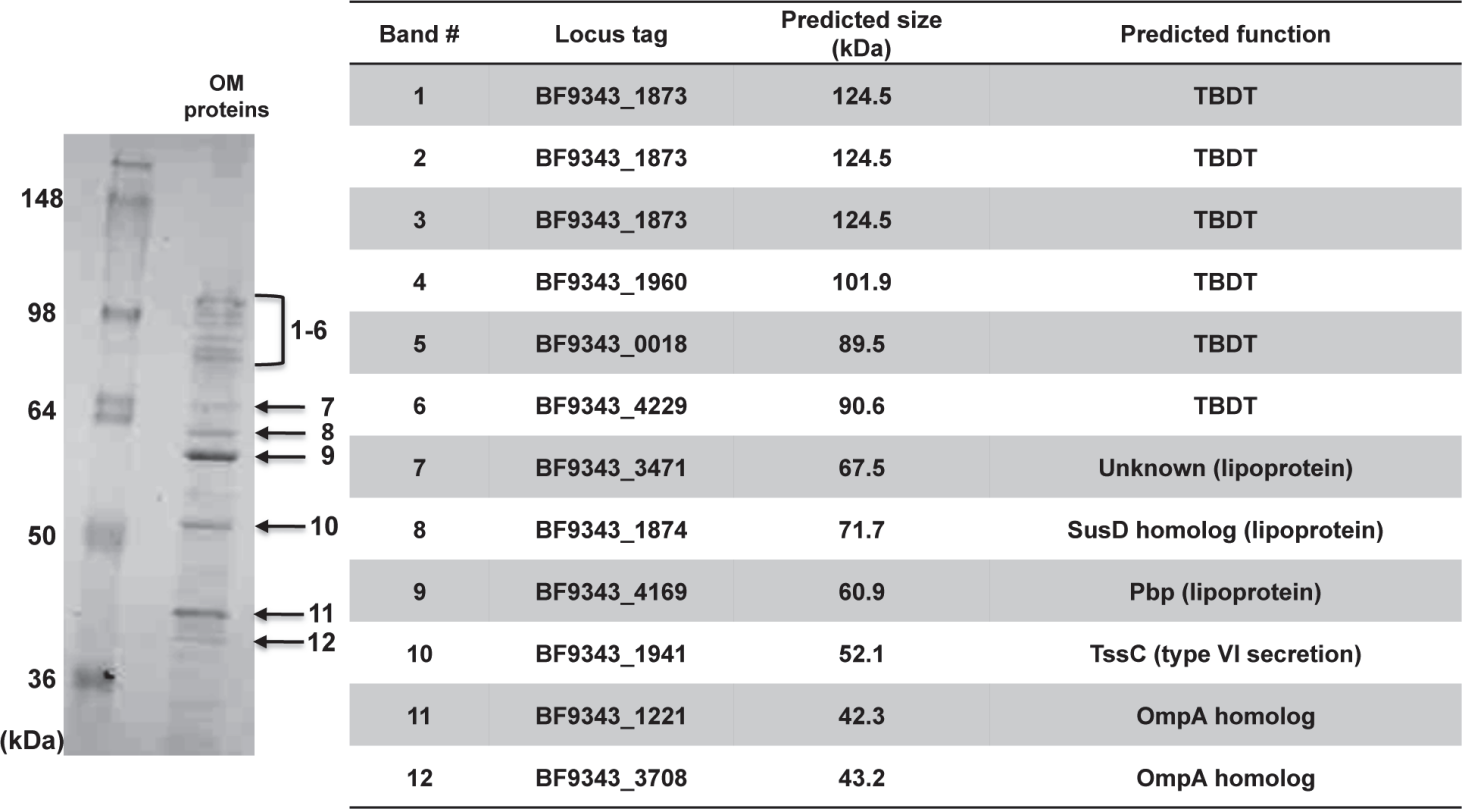
Identification of highly expressed OM proteins. An OM fraction was subjected to SDS-PAGE, and proteins were visualized by Colloidal Blue staining. The indicated bands were excised from the gel and analyzed by LC/MS/MS. The proteins that are most likely contained in each band and their predicted functions are listed in the table.

We next identified OM proteins that are highly abundant by subjecting an OM fraction to one-dimensional SDS-PAGE. The most prominent Colloidal blue-stained bands were excised, and the proteins in each band were identified by mass spectrometry (Fig. 3 and S3 Table). Two of the proteins that we found are OmpA-like proteins. One of these proteins, BF9343_3708, was stained relatively lightly but was previously described as the most abundant OM protein of *B. fragilis* (Fig. 3, band 12) [57]. A second OmpA-like protein, BF9343_1221, has been shown to be expressed using RT-PCR [22] and stained darkly (Fig. 3, band 11). We also identified several high molecular weight (~90–125 kDa) proteins that correspond to TBDTs (Fig. 3, bands 1–6). The abundance of these proteins is striking given that the expression of *E. coli* TBDT genes is tightly regulated and linked to the concentration of substrate molecules [58]. Presumably because the TBDTs were not well resolved, the highest probability candidate for three of the bands is BF9343_1873 (*omp121*). This protein has been shown to form the *omp200* porin complex together with the SusD homolog BF9343_1874 (*omp71*) [15], a lipoprotein that was also a prominent band on our gels (Fig. 3, band 8). In addition to BF9343_1874, we identified two other lipoproteins, the previously described plasminogen-binding protein BF9343_4169 (Pbp) [19], which was the most darkly stained OM protein, and BF9343_3471, a lipoprotein of unknown function that is predicted to have xylanase and lipocalin_6 domains (Fig. 3, bands 7 and 9). The overall profile was consistent with that observed in a recent study [13].

**Fig 4:**
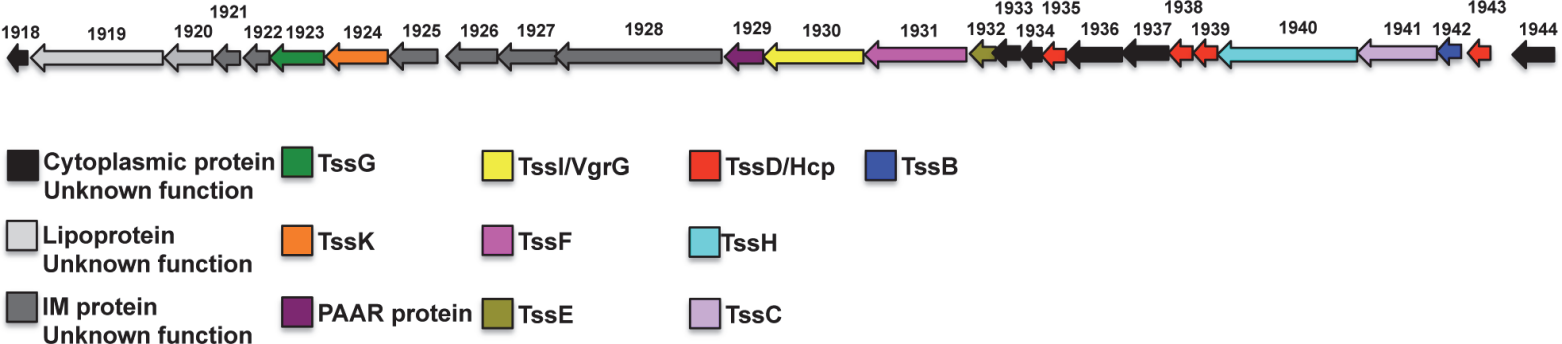
Genetic organization of the putative type VI secretion locus of *B. fragilis*. The identity of individual components of the putative type VI secretion system is based on their homology to components of another putative type VI secretion system that is encoded in the *Bacteroides cellulosilyticus* CL02T12C19 genome [81].

Interestingly, we also identified a homolog of a protein that forms part of the sheath structure of the Proteobacterial type VI secretion apparatus (TssC) in our analysis of abundant OM proteins (Fig. 3, band 10). This protein (BF9343_1941) is encoded in a large gene cluster that appears to encode most of the structural components of a type VI system (Fig. 4). This locus was recently identified in the genomes of *B. fragilis* and other *Bacteroides* [33,59]. Genes encoding a few known IM components seem to be missing from the locus, but may have escaped detection simply because the protein sequences have not been well conserved. Curiously, BF9343_1941 lacks a signal peptide, and TssC is thought to be a cytoplasmic component of the type VI machinery in Proteobacteria [60]. Presumably the protein was isolated in the OM fraction because it is associated with a macromolecular complex that traverses the OM. One other protein that is encoded in the same locus (BF9343_1942) appeared on our original list of 328 potential OM proteins, but was eliminated because it lacks a signal peptide (S1 Table). An additional protein encoded in this locus (BF9343_1943) is a homolog of the TssD tube protein and was found to be an abundant component of the *B. fragilis* secretome (see below). The discovery that TssC and TssD homologs are produced at a high level by *B. fragilis* grown in a monoculture is striking given that the production of type VI secretion systems by Proteobacteria is often tightly regulated and induced by specific signals [60].

### A high percentage of lipoproteins are cell surface-exposed

To identify proteins that are exposed on the cell surface, intact cells were treated with PK or left untreated, and proteins in each sample were detected using shotgun proteomics. Label-free quantification was used to assess changes in the level of each protein. As in the experiments described above, only proteins that were represented by at least four unique peptides were considered. A protein was defined as PK-sensitive if its level was reduced at least five-fold by protease treatment. As a control, the experiment was repeated, but a purified membrane fraction obtained from untreated cells was also incubated with PK to digest proteins that reside on both sides of the OM. The majority of OM proteins that were found to be PK- resistant in intact cells were PK-sensitive in the membrane fraction (S1 and S2 Tables), indicating that they are not inherently PK-resistant. In the control experiment, the PK treatment of intact cells yielded results that were largely consistent with those of the initial screening, although a direct comparison could not be made because fewer proteins were identified.

Only about 25% of the β barrel proteins were sensitive to proteolysis when intact cells were treated with PK. The PK-sensitive group included BF9343_2862, which like other members of the type Vd secretion family would be expected to have a large extracellular domain. In contrast, BamA was only digested when the periplasmic side of the OM was exposed to PK (S1 Table). This finding is consistent with the observation that at least under some conditions E. coli BamA is resistant to PK digestion [61]. Most of the predicted efflux pumps and the polysaccharide transporter Wza were also PK-resistant, presumably because they contain only very small segments that are exposed on the cell surface. Like SusC [62], most of the TBDTs that we identified were resistant to PK digestion. Although crystallographic analysis has shown that TBDTs can have large extracellular loops [58], our results suggest that exposed segments of B. fragilis TBDTs generally fold into a compact, protease-resistant structure. When a purified membrane fraction was treated with PK, the majority of the TBDTs were sensitive to digestion (S1 Table).

**Fig 5:**
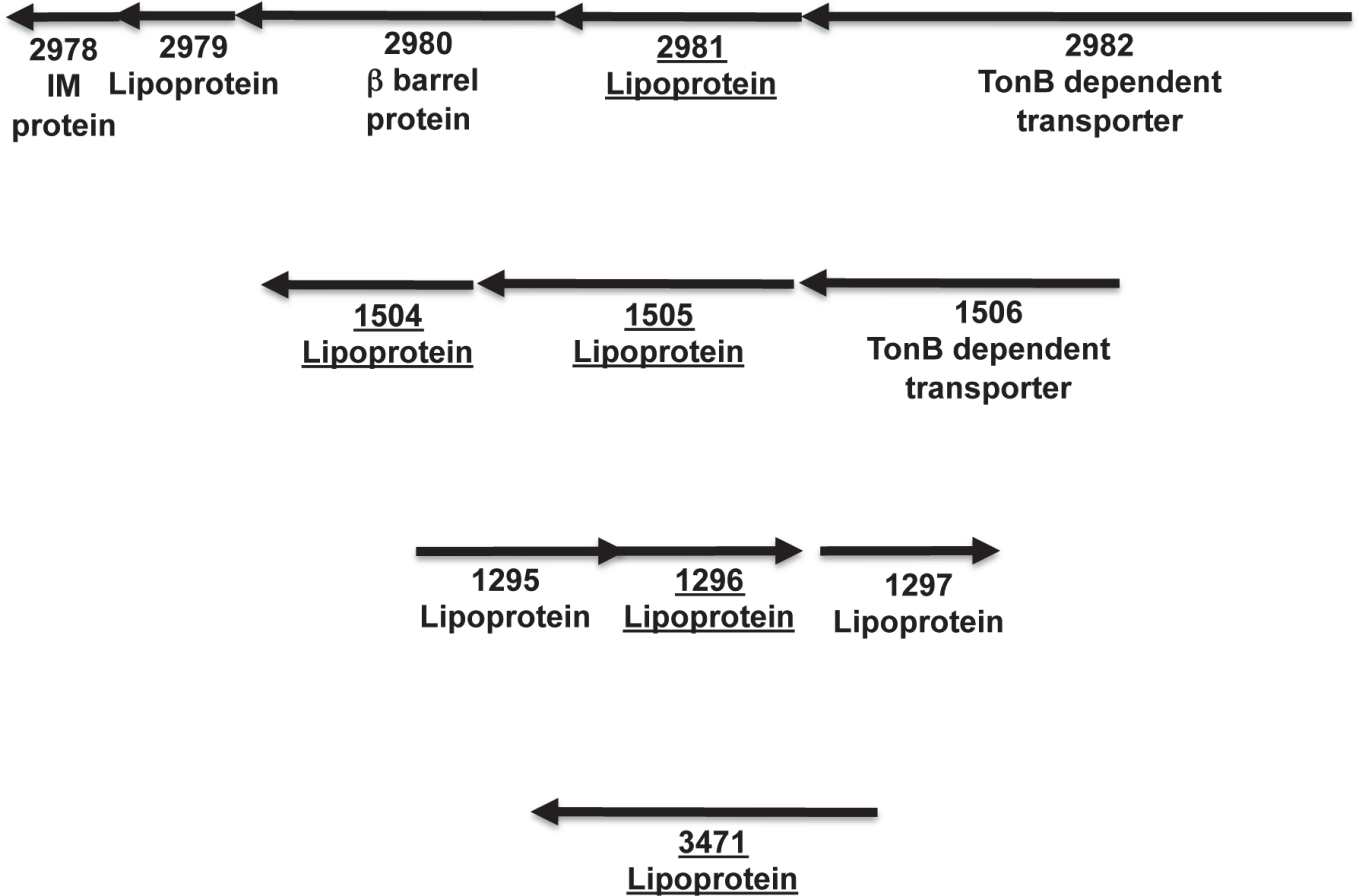
Examples of the chromosomal context of genes encoding PK-sensitive lipoproteins. Genes that are underlined encode proteins that were both PK-sensitive and biotinylated. For simplicity, only the numerical portion of the locus tag (i.e., the four numbers following BF9343_) is shown.

**Fig 6:**
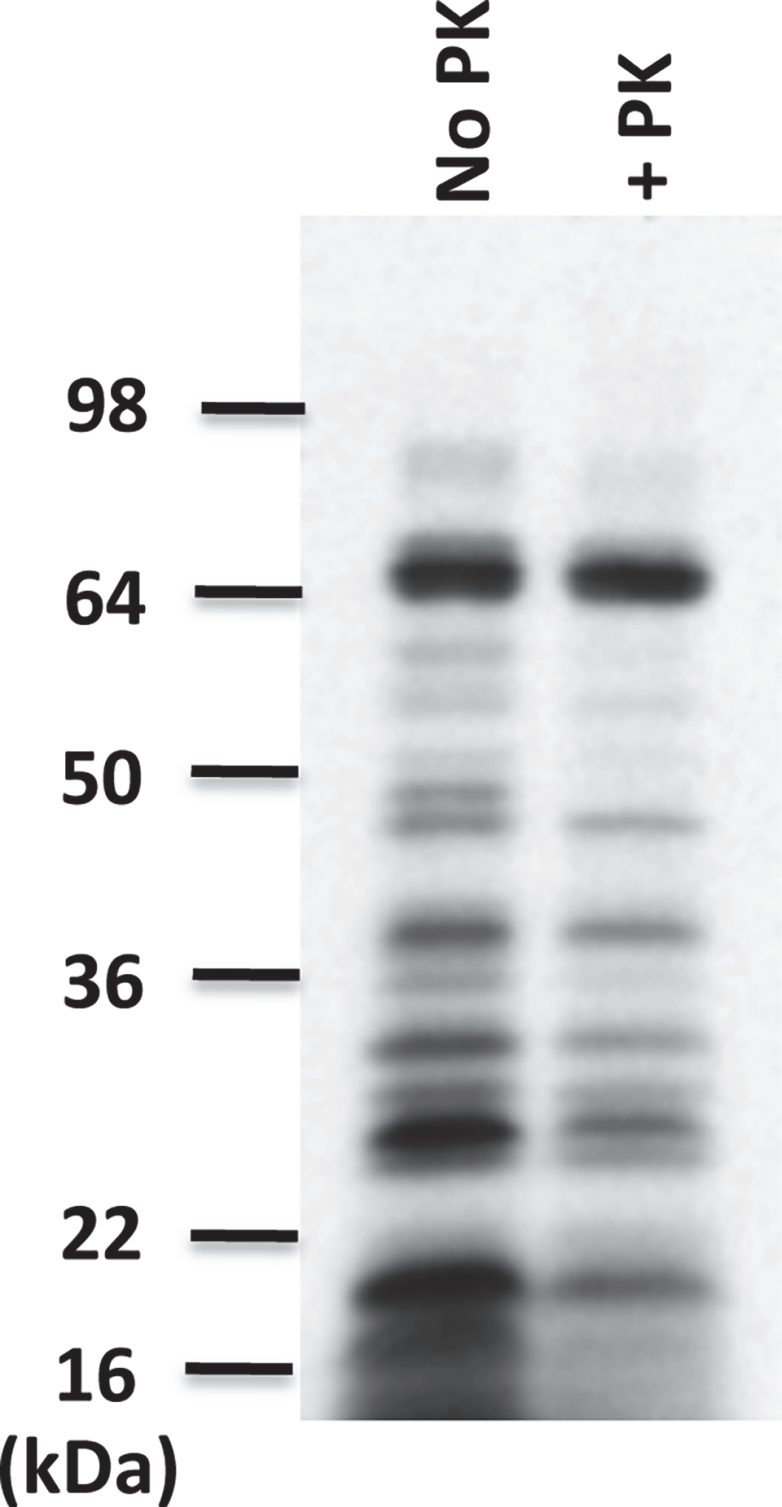
A large fraction of *B. fragilis* lipoproteins are sensitive to PK digestion. Cells were incubated with ^3^H-palmitic acid and divided in half. One half was treated with PK, and OM fractions were prepared from both treated and untreated cells. Radiolabeled proteins were then separated by SDS-PAGE and visualized using fluorography.

Interestingly, although only a small number of surface-exposed lipoproteins have been described in Gram-negative bacteria, a significant proportion of the lipoproteins we identified (38%) were sensitive to PK treatment (Fig. 1 and S1 Table). Nine of the PK-sensitive lipoproteins are SusD homologs and 18 others are encoded adjacent to TBDT genes on the *B. fragilis* chromosome (Figs. 1 and 5 and S4 Table). These proteins presumably play a role in nutrient acquisition analogous to the Sus lipoproteins [2]. Indeed BF9343_0985 is homologous to the heme-binding lipoprotein HmuY of Porphyromonas gingivalis, which is thought to act at the cell surface in conjunction with the TBDT HumR to import heme [63]. Several other PK-sensitive lipoproteins are not encoded near TBDT genes, however, and three of them appear to be encoded in a single operon (Fig. 5). The observation that BF9343_3471 and AapA (BF9343_3703) were PK-sensitive is consistent with other evidence that they are exposed on the cell surface [20], although AapA was not included in the final results because we could not identify at least 4 unique peptides. Curiously, two lipoproteins that were previously reported to be PK-sensitive, Pbp [19] and the glycoprotein BF9343_0498 [21], were resistant to PK digestion in our hands. At least in the case of Pbp, this disparity might be due to the fact that we used a different *B. fragilis* strain. To confirm that the surface exposure of lipoproteins is a widespread phenomenon in *B. fragilis*, we incubated cultures with ^3^H-palmitic acid and treated half of the cells with PK. Whole cell extracts were then subjected to SDS-PAGE and radiolabeled lipoproteins were visualized by fluorography. As expected, the level of a large fraction of the proteins was strongly reduced by PK treatment (Fig. 6).

To further explore the spectrum of proteins that are exposed on the cell surface, we next treated intact cells with a lysine-specific membrane-impermeable biotinylation reagent (Sulfo-NHS-SS-Biotin), isolated biotinylated proteins by affinity chromatography using NeutrAvidin agarose, and identified the purified proteins using shotgun proteomics. This approach not only provided a method to confirm the localization of proteins that were PK-sensitive, but also provided a means of identifying proteins that contain exposed segments but that are either inherently resistant to PK treatment or protected from PK digestion through an interaction with another protein. SusD, for example, is thought to act at the cell surface, but is resistant to PK treatment unless SusC is absent [62]. To minimize the background of contaminating proteins that lack signal peptides, we analyzed only the 213 proteins that were represented by 12 or more unique peptides (S5 Table; proteins that fell below the cutoff are listed in S6 Table). This dataset was then further subdivided based on the percentage of identified peptides that contained a modified lysine residue.

Based on the degree of biotinylation, many of the proteins that are likely to be exposed on the cell surface are lipoproteins. Thirteen of the 18 very highly biotinylated proteins (≥ 50% biotinylated peptides) and 8 of the 13 highly biotinylated proteins (40–49% biotinylated peptides) were lipoproteins. One of the most highly biotinylated proteins (BF9343_0962) is a predicted β barrel protein of unknown function. With one exception, all of the other very highly or highly biotinylated proteins contain signal peptides or are predicted IM proteins. Almost all of the TBDTs and six of the SusD homologs we identified [including BF9343_1638, a protein that was recently localized to the cell surface based on immunofluorescence [64]], were only modestly biotinylated (≤20% biotinylated peptides). The other six SusD homologs we identified showed only slightly higher levels of biotinylation (20–29% biotinylated peptides). The results suggest that the extracellular lysines of TBDTs and their associated lipoproteins were not accessible to the biotinyation reagent and are consistent with other evidence that these proteins form a tightly folded complex. Furthermore, the observation that multiple OMPs that are known to have only small exposed segments in other bacteria (e.g., TolC-like molecules, BamA and Wza) were only modestly biotinylated helped to validate the biotinylation assay as a means of determining protein localization.

The data from the PK and biotinylation experiments were combined to give a list of 18 high confidence surface-exposed proteins (Table 1). The proteins on this list were either PK- sensitive and highly biotinylated, or PK-resistant and very highly biotinylated. The fact that almost all of the proteins are predicted to be lipoproteins corroborates our conclusion that there is extensive cell surface localization of this class of proteins in *B. fragilis*. About half of the lipoproteins are encoded adjacent to genes that encode TBDTs and therefore may be involved in nutrient acquisition. Surprisingly, one of the proteins that was both sensitive to PK digestion and very highly biotinylated is SurA, a chaperone that has been shown to promote the assembly of OMPs in *E. coli* [55]. While the accessibility of SurA to proteases and chemical modifying reagents may be an artifact of its high concentration in the periplasm, the possibility that it is at least transiently exposed on the cell surface in *B. fragilis* cannot be ruled out.

**Table 1:** High confidence surface-exposed proteins^1^.

Locus tag	Amino acids	Unique modified peptides^1^ / % modified peptides	PK sensitive	Type	Predicted Function^2^
BF9343_4169	559	41 / 65%	No	Lipoprotein	Pbp
BF9343_3471	623	20 / 50%	Yes	Lipoprotein	Unknown*
BF9343_4195	287	17 / 68%	No	Lipoprotein	Putative FKBP-type peptidyl-prolylcis-trans isomerase
BF9343_2074	472	14 / 52%	Yes	Lipoprotein	Unknown*
BF9343_3356	400	13 / 50%	Yes	Lipoprotein	Unknown*
BF9343_0559	285	12 / 60%	No	Lipoprotein	Unknown
BF9343_3784	456	11 / 52%	Yes	Periplasmic protein	SurA
BF9343_1466	463	10 / 56%	Yes	Lipoprotein	Unknown
BF9343_2979	194	10 / 67%	No	Lipoprotein	Unknown*
BF9343_3541	145	10 / 83%	No	Lipoprotein	Unknown
BF9343_2956	267	9 / 53%	Yes	Lipoprotein	Unknown (NigD-like)
BF9343_2076	215	9 / 60%	NF	Lipoprotein	Unknown*
BF9343_2621	283	8 / 47%	Yes	Lipoprotein	Unknown*
BF9343_0962	351	8 / 57%	NF	β barrel protein	Unknown
BF9343_2991	198	7 / 54%	No	Lipoprotein	Unknown (Skp-like)
BF9343_1589	165	7 / 50%	No	Lipoprotein	Unknown
BF9343_1504	455	6 / 43%	Yes	Lipoprotein	Unknown*
BF9343_2981	404	5 / 42%	Yes	Lipoprotein	Unknown*

^1^Proteins listed are either PK-sensitive and highly biotinylated (>40% biotinylated peptides) or PK-resistant and very highly biotinylated (>50% biotinylated peptides). Three very highly biotinylated proteins that are predicted to be localized in the cytoplasm or IM were excluded from the list. NF = not found in experiments in which PK-sensitivity was assessed.

^2^Peptides found to have a CAMthiopropanoyl-modified Lys residue. The modification resulted from the cleavage of the biotinylation reagent that was bound at this position.

^3^Function was predicted using BLAST searches and information in the NCBI Conserved Domain Database. Lipoproteins encoded near or adjacent to TBDT genes are denoted by an asterisk.

### Multiple potential type I secretion systems are produced by *B. fragilis*

Whereas *E. coli* produces a single OM protein called TolC that functions in conjunction with a wide variety of IM transporters and adaptor proteins to export both small hydrophobic molecules and proteins, we found that the *B. fragilis* genome encodes >20 putative TolC homologs. All of these homologs are encoded adjacent to genes that are predicted to encode either a multidrug resistance (MDR) pump or an ABC transporter and a so-called membrane fusion protein (MFP). MFPs are typically lipoproteins that are anchored to the IM [65] or single-pass IM proteins with large periplasmic domains that link the transporters to TolC. Remarkably, we found that 12 of the TolC-like proteins are produced when cells are grown in TYG (S7 Table). We also identified eight of the corresponding MFPs, but it is unclear whether these proteins were isolated because they are bona fide components of the OM proteome or because they are bound tightly to TolC homologs.

**Fig 7:**
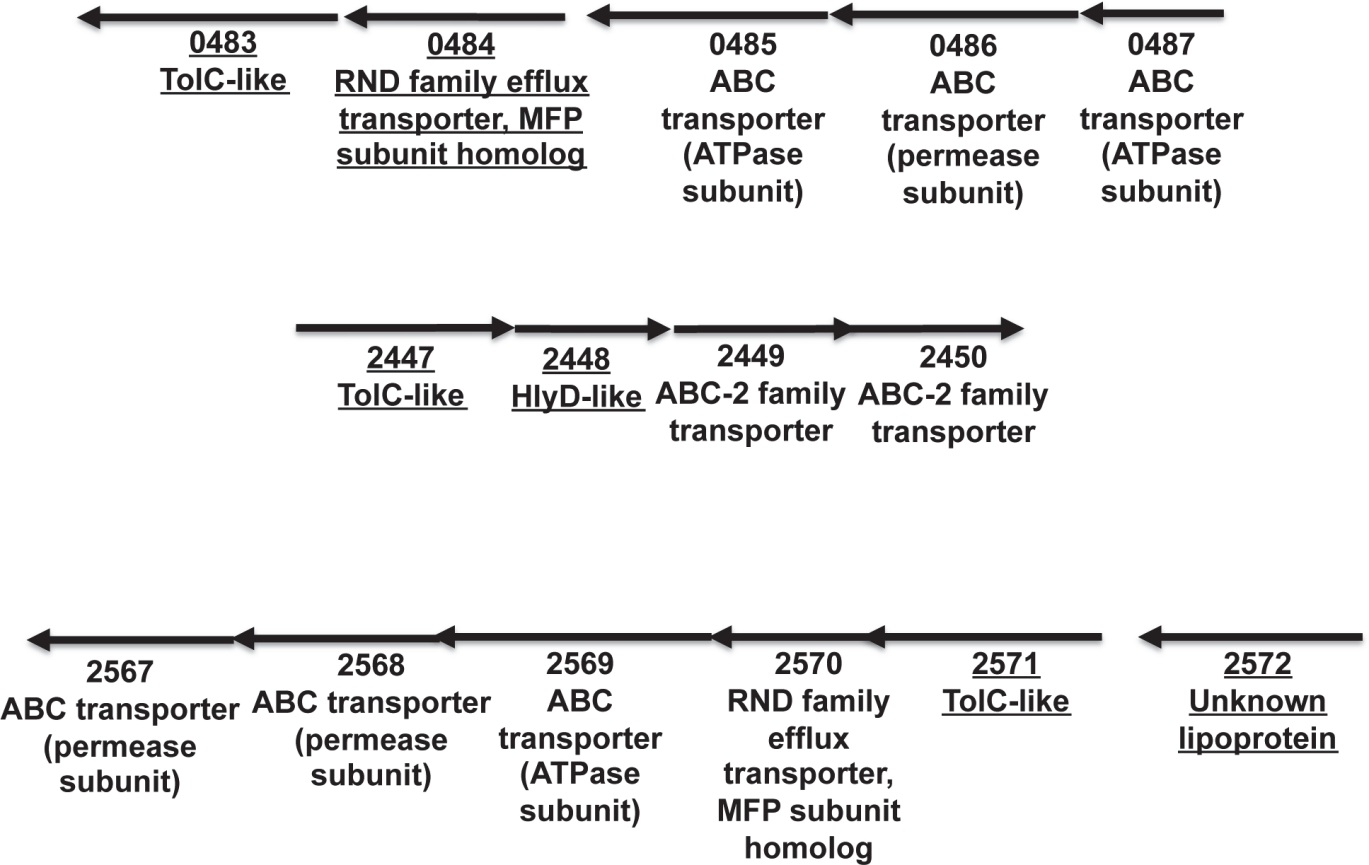
Organization of *B. fragilis* loci encoding putative type I secretion systems expressed under laboratory conditions. Genes encoding proteins that were identified by LC-MS/MS analysis are underlined. For simplicity, only the numerical portion of the locus tag (i.e., the four numbers following BF9343_) is shown.

The majority of the TolC-like proteins are encoded adjacent to MDR pumps and presumably function in multidrug resistance. Consistent with this prediction, the disruption of a gene that encodes the putative MDR pump OprM (BF9343_2859) has been shown to increase sensitivity to specific antibiotics [66]. Three of the TolC-like proteins, however, are encoded adjacent to genes that encode ABC transporters and are therefore predicted to be components of type I secretion systems or related secretion systems such as the *E. coli* enterotoxin exporter MacAB (Fig. 7). In each case an MFP or lipoprotein encoded in the same locus was also identified. Like the MFP component of other type I secretion systems, two of the three MFPs are predicted to be single-pass IM proteins. Interestingly, homology searches revealed that the *B. fragilis* genome contains at least four additional loci that appear to encode all three components of type I secretion systems (S8 Table). Curiously, the substrates of this multitude of putative type I secretion systems are not obvious because the C-terminal GGXGXDXXX sequence that functions as a targeting signal for type I secretion in Proteobacteria and Cyanobacteria [65] is not present in any *B. fragilis* proteins.

### Conservation of the OM proteome in cells grown in different carbon sources

**Fig 8:**
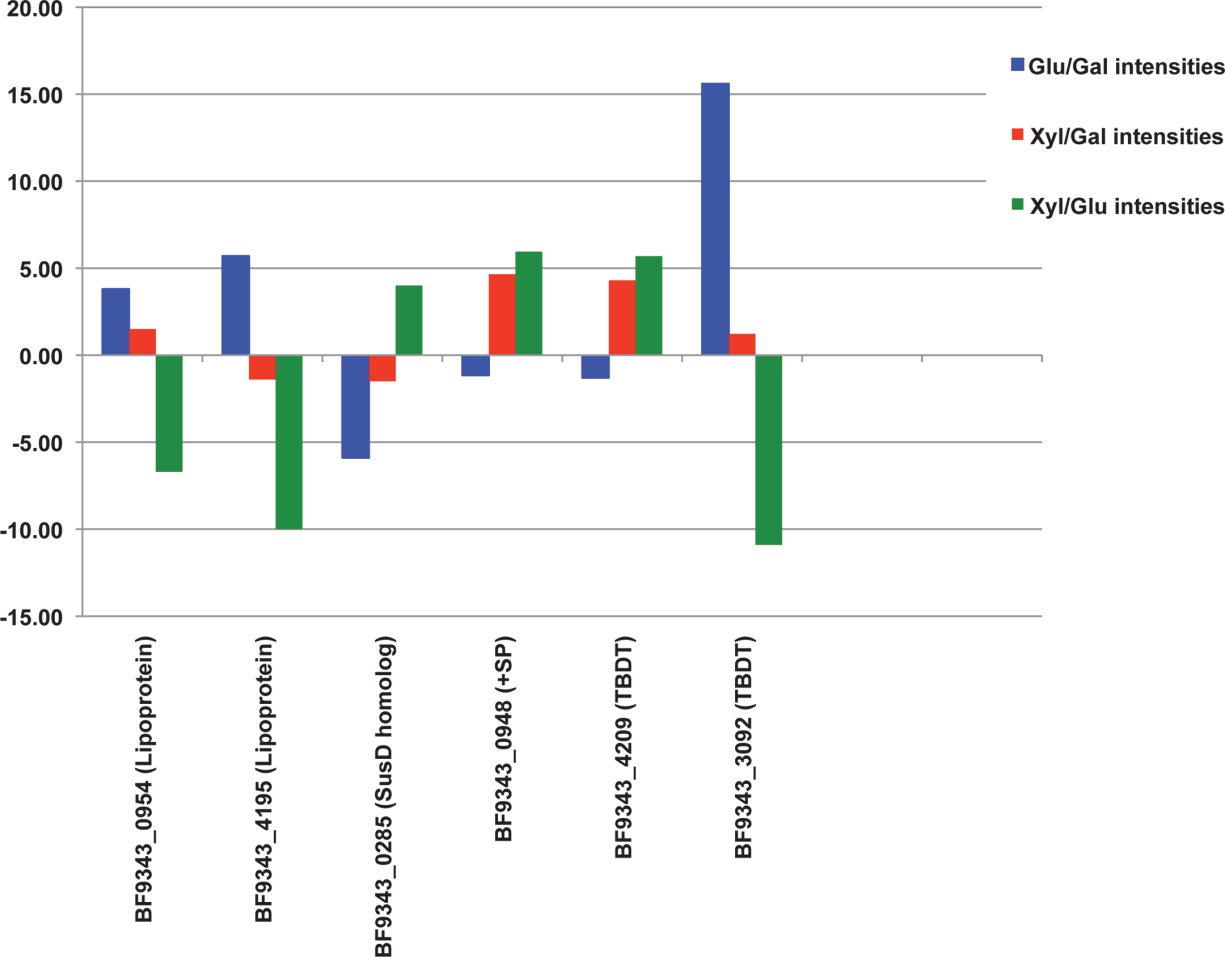
The level of only a few OM proteins varies significantly upon growth in different media. Proteins that have a significant difference in MS intensities (at least 5-fold higher or lower) in defined minimal medium containing glucose (Glu), galactose (Gal) or xylose (Xyl) as the sole carbon source are shown. +SP denotes a signal peptide-containing protein that is not predicted to be either a lipoprotein or a β barrel protein.

Because genes that encode TBDTs in *E. coli* are generally expressed at a very low level except in the presence of their small molecules substrates, we were surprised to find that a large number of *B. fragilis* TBDTs were simultaneously produced at a high level when cells were grown in TYG. To examine the expression of genes that encode TBDTs and other OMPs under different nutritional conditions, cells were grown in defined minimal media containing glucose, galactose or xylose as the sole carbon source. The level of OMPs produced under each condition was then compared by quantitative mass spectrometry using stable isotope dimethyl labeling. Interestingly, the level of only six out of 131 putative OMPs that were identified showed a change of >5-fold between the samples (Fig. 8 and S9 Table). Furthermore, many of the TBDTs that were identified in cells grown in TYG were also produced at a high level in cells grown in the defined media irrespective of the carbon source. While these results indicate that monosaccharides do not strongly influence the expression of genes encoding a core set of TBDTs and other OM proteins, previous studies have suggested that the presence of different complex carbohydrates, iron limitation and the state of anaerobiosis can significantly alter the protein content of the OM [38,67,68]. Thus oligosaccharides or other molecules may play a more important role in regulating the synthesis of OM transporters in *Bacteroides* than monosaccharides.

### Composition of the *B. fragilis* secretome

**Fig 9:**
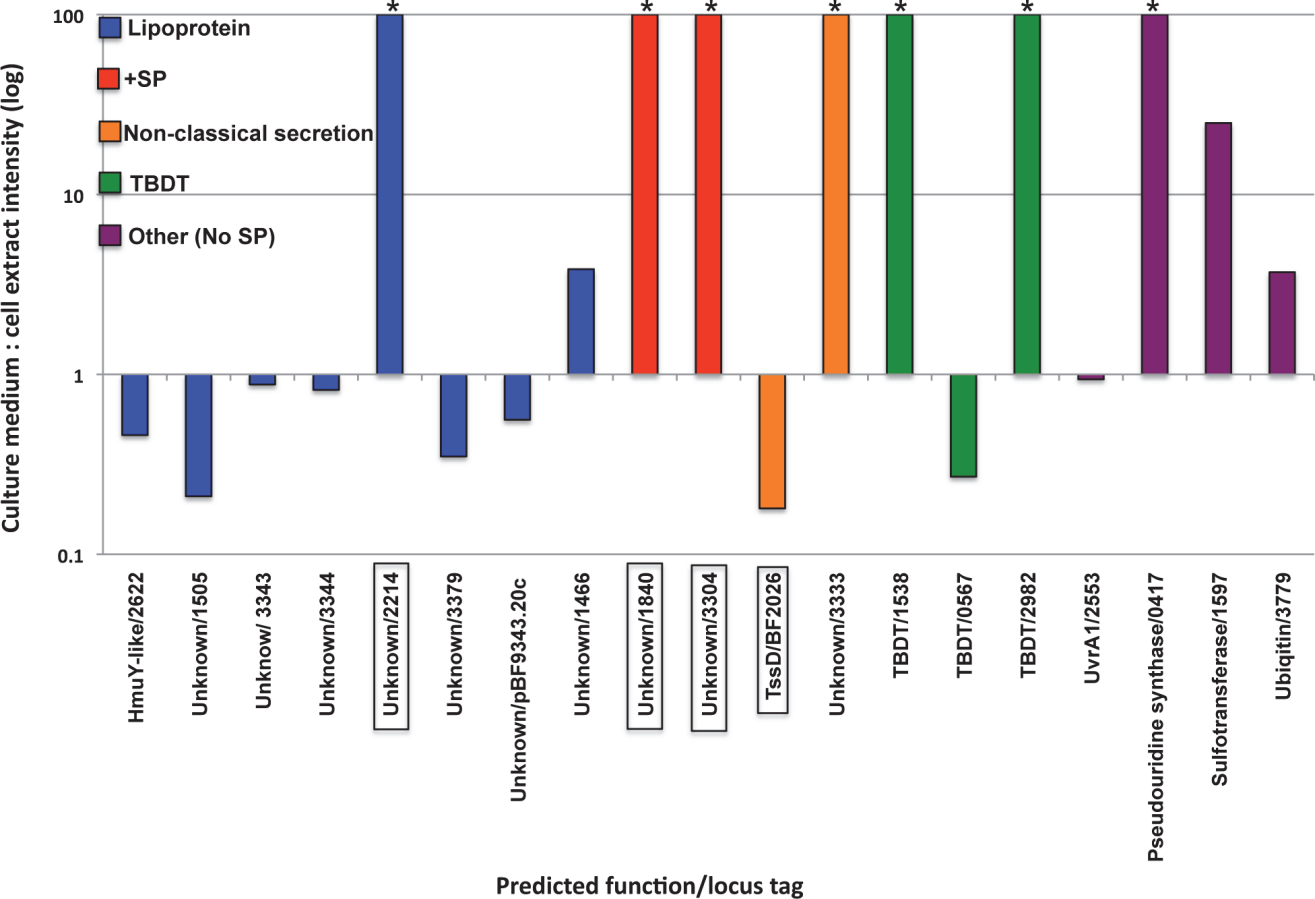
Composition of the *B. fragilis* secretome. Proteins that were most highly enriched in the culture medium (based on the relative intensity of the LC-MS/MS signal in the culture supernatant and whole cell extract) are shown. Proteins designated with an asterisk were found exclusively in the culture medium. Proteins that are boxed were not found in our analysis of the OM proteome. +SP denotes a signal peptide-containing protein that is not predicted to be either a lipoprotein or a β barrel protein. For simplicity, only the numerical portion of the locus tag (i.e., the four numbers following BF9343_) is shown.

To identify proteins that are secreted into the extracellular milieu, we first removed cells from actively growing cultures by centrifugation. To exclude any OM vesicles that might have been released from the cells, the resulting supernatant was centrifuged at high speed and the pellet was discarded. A total of 42 proteins were identified in the culture medium (S10 Table). About half of these proteins are very abundant cytoplasmic proteins including EF-Tu, the ribosomal protein L12 and enolase that were presumably released into the culture medium upon the lysis of a small fraction of the cells. To exclude these proteins from our analysis, we determined the ratio of each protein in the culture supernatant relative to a whole cell extract using stable isotope dimethyl labeling followed by quantitative LC-MS/MS. As expected, most of the cytoplasmic proteins were found predominantly in the cell extract (culture medium: cell extract ratio <0.1). Some proteins, however, might be secreted slowly and therefore present at a relatively high level in the cell extract. To account for this possibility, we considered the proteins that were the most highly enriched in the culture medium (culture medium: cell extract ratio >0.18) to be the most likely components of the secretome (Fig. 9).

Only one of the 19 proteins that met our criteria, the ubiquitin homolog BF9343_3779, has been previously isolated in the culture medium of *B. fragilis* [69]. Seven proteins were found almost exclusively in the culture medium (Fig. 9, asterisks). This group includes four proteins that did not appear in our analysis of the OM proteome (Fig. 9, boxes). Eight of the 19 proteins are predicted to be lipoproteins. Other than BF9343_2622, which is homologous to the TBDT-associated protein HmuY, and BF9343_3703 (AapA), which is a surface exposed protein that may play a role in adherence [20], none of these lipoproteins can be easily classified. It should be noted that based on our proteomic analysis alone we cannot determine whether the entirety of these lipoproteins was present in the medium or only a fragment that was released from the cell surface by a proteolytic cleavage. Two of the proteins we identified (BF9343_1840 and BF9343_3304) contain conventional signal peptides. BLAST searches revealed that the *B. fragilis* genome also encodes three closely related proteins (BF9343_2361, BF9343_2081, and BF9343_1558). Although the function of this family of proteins is unknown, they range in size from 104–112 amino acids and are found almost exclusively in the phylum *Bacteroidetes*. Curiously, the other six proteins lack signal peptides. One of these proteins (BF9343_1943) is a homolog of the TssD protein that forms the extracellular tube structure of the type VI secretion apparatus. This protein has been found in the culture medium of other bacteria [60]. A second protein is predicted to be secreted by a non-classical pathway [48]. The other proteins that lack signal peptides are predicted to be TBDTs or are related to cytoplasmic proteins (such as ubiquitin).

While the sequences of the proteins in the secretome do not reveal any clues about the mechanism by which they are secreted, some of them may be substrates of the putative type I secretion systems described above. It is also conceivable that some of them are substrates of the type VI secretion system, although proteins that are secreted by cognate systems in Proteobacteria are generally injected directly into target cells and are not found in the environment [60]. In principle, the proteins that lack signal peptides could be transported from the cytoplasm to the extracellular space through either the type I or type VI pathway. In contrast, the small signal sequence-containing proteins might first be translocated into the periplasm by the Sec pathway and then targeted to an ABC transporter/TolC-based export system, like the enterotoxins produced by *E. coli* [70].

**Fig 10:**
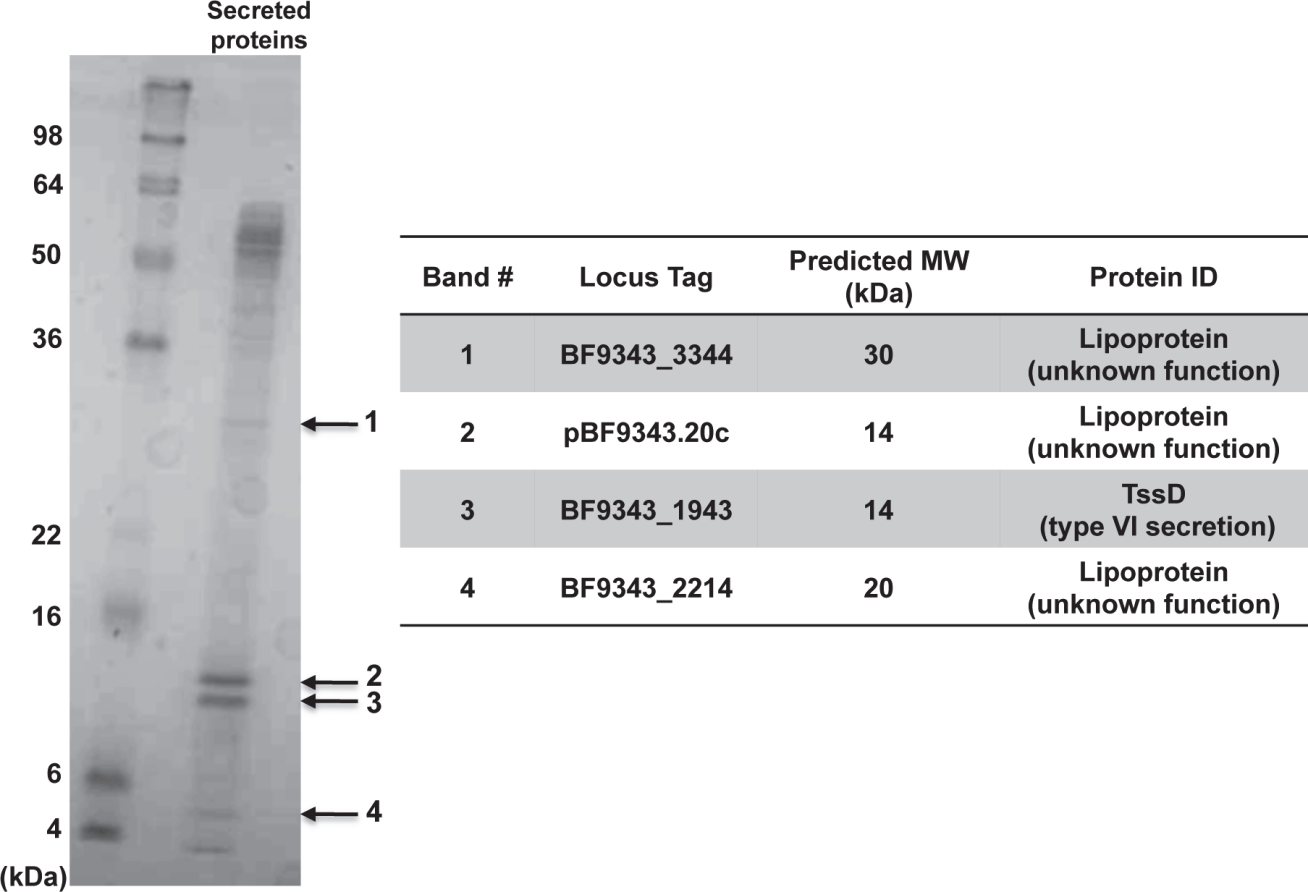
Identification of highly expressed secreted proteins. Proteins that were present in the culture medium were subjected to SDS-PAGE and visualized by Colloidal Blue staining. The indicated bands were excised from the gel and analyzed by LC-MS/MS. The proteins that are most likely contained in each band are listed in the table.

Finally, we analyzed the culture medium by one-dimensional SDS-PAGE to identify the most abundant constituents of the secretome. The results indicate that the culture medium is enriched in 50–60 kDa proteins and small proteins (<20 kDa) (Fig. 10 and data not shown). Four of the most prominently stained bands were excised from the gel and identified by mass spectrometry (Fig. 10 and S11 Table). All of these proteins were among the 19 proteins described above that were identified as likely components of the secretome. Three of them are lipoproteins of unknown function, while the fourth is the TssD homolog. The finding that the homologs of TssC and TssD are among the most abundant components of the OM proteome and secretome, respectively, suggests that the copy number of type VI secretion complexes is relatively high.

## DISCUSSION

In this study we describe the first analysis of the OM proteome and secretome of a *Bacteroides* species. We found that the composition of the OM proteome of *B. fragilis* grown in standard laboratory media differs considerably from that of well studied “model” Gram-negative bacteria such as *E. coli* K-12. Whereas porins such as OmpA and OmpC are the predominant components of the *E. coli* OM [71], we found that TBDTs and lipoproteins are the major constituents of the *B. fragilis* OM. Furthermore, we found not only that the *B. fragilis* genome is predicted to encode a much greater diversity of TBDTs (92 vs. 9; ref. 10) and lipoproteins (177 vs. 86; ref. 16) than the *E. coli* genome, but that a large fraction of the predicted TBDTs and lipoproteins are produced at detectable levels under standard laboratory conditions. While protease accessibility or biotinylation assays are typically used to assess the surface exposure of OM proteins [72–74], we used a combination of these approaches to obtain a more complete and accurate data set. Surprisingly, the results revealed that a high percentage of the lipoproteins are cell surface-exposed. A variety of lipoproteins were also found in the secretome. Other constituents of the secretome included two ~100 residue signal peptide-containing proteins that appear to be members of a conserved protein family, several proteins that lack signal peptides, and three TBDTs that are not readily distinguishable from the large number of TBDTs that reside in the OM. While all of these proteins were highly enriched or found exclusively in the culture medium, neither their function in the extracellular space nor the mechanism by which they are secreted is readily apparent. Curiously, none of the secreted proteins (other than the TBDTs) are predicted to facilitate nutrient uptake or mediate antimicrobial activities. A recent study indicates that *B. fragilis* also preferentially packages specific proteins into OM vesicles that are shed from the cell surface [13], but we did not analyze the contents of these vesicles here.

It is striking that *B. fragilis* synthesizes a large number of TBDTs and lipoproteins that likely function in conjunction with them even in a very simple nutritional environment. Consistent with our results, an analysis of the transcriptome of *B. thetaiotaomicron* indicated that the expression of many TBDT genes is constitutive [75]. Based on their size and the genomic context of the genes that encode them, the TBDTs that we identified fall into two categories. A majority (39 out of 61) range in size from 998–1138 amino acids and are encoded adjacent to genes that encode SusD homologs. These TBDTs presumably mediate the uptake of a wide variety of dietary and host-derived polysaccharides that *B. fragilis* can metabolize. In many cases genes encoding additional lipoproteins, glycosyl hydrolases or other enzymes that are presumably the counterparts of the SusA, SusB, and SusE-G proteins involved in starch utilization are found in the same locus. We also identified a second group of smaller TBDTs (678–929 amino acids in size) that are not encoded adjacent to genes encoding SusD homologs. These TBDTs have conserved domains that suggest that they are involved in the uptake of substrates such as iron siderophores and cobalamin. Indeed *E. coli* TBDTs that transport these types of substrates (including BtuB, CirA, TbpA, FepA, FhuA, FecA and HmbR) are similar in size (614–915 amino acids). In any case, the finding that *B. fragilis* produces a large number of TBDTs constitutively implies that it utilizes its resources differently from *E. coli*, which tends to produce proteins upon demand. For reasons that are unclear, this strategy may help *B. fragilis* adapt more rapidly to changes in the availability of polysaccharides in the gut. It was recently shown, however, that the synthesis of a TBDT and other proteins required for chondroitin sulfate (CS) utilization in *B. thetaiotaomicron* increases dramatically upon exposure to a CS breakdown product [76]. This observation suggests that the expression of at least some *Bacteroides* genes is altered in response to nutritional cues.

While the cell surface localization of a few lipoproteins has been observed in a variety of Gram-negative bacteria, no organism has been shown to export as many lipoproteins as *B. fragilis*. Given that the surface exposed lipoproteins are a structurally and functionally diverse group of proteins that do not contain an obvious targeting signal, the mechanism of export is unclear. While it is tempting to speculate that the lipoproteins are first targeted to the OM and then transported across the lipid bilayer, it is also possible that they are secreted into the medium and then reattached to the cell surface. Studies in *B. burgdorferi* have shown that an N-terminal peptide derived from a surface exposed lipoprotein promotes efficient secretion of a reporter protein [77]. Perhaps N-terminal peptides play a similar role in dictating the localization of lipoproteins in *B. fragilis*. While the factors that promote surface exposure of lipoproteins are unknown, it is possible that the Bam complex, which catalyzes the membrane integration of β barrel proteins, plays a role in the process. It is also conceivable that lipoproteins are exported by multiple mechanisms in *B. fragilis*. The export of lipoproteins that are associated with TBDTs might, for example, be coordinated with the membrane integration of the cognate TBDT, while lipoproteins that function independently are targeted to a dedicated transport machine.

Our results imply that the secretion pathways that predominate in *B. fragilis* are very different from those that predominate in *E. coli* and related Proteobacteria. In addition to making extensive use of a lipoprotein export pathway, *B. fragilis* also secretes several patatin-like polypeptides by the type Vd pathway. Curiously, the other type V pathways that are widespread in Proteobacteria (type Va-c and type Ve) appear to be absent. Furthermore, the *B. fragilis* genome encodes seven putative type I secretion systems (three of which are expressed under laboratory growth conditions), while the *E. coli* genome encodes only one type I secretion system. Like many Proteobacteria, *B. fragilis* also produces a type VI secretion system. While the function of this multiplicity of secretion machines is unclear, it is possible that they promote the cell surface localization of lipoproteins and/or the export of at least some of the proteins we identified in the secretome. In any case, the absence of the type II, III and IV and most of the type V pathways that are prevalent in Proteobacteria is intriguing. Perhaps there is a mechanistic or assembly barrier that selects against the production of these secretion systems in *B. fragilis*. Alternatively, *B. fragilis* may not produce the types of proteins that are secreted through these pathways. Indeed many proteins that are secreted by the type II-type V pathways have virulence functions that might not be needed by a predominantly commensal organism. Although type VI systems are also often associated with virulence, *B. fragilis* appears to use this pathway to secrete antimicrobial factors that reduce competition [78]. One indication that secretion pathway utilization is affected by phylogenetic barriers emerges from an analysis of OM and secreted proteins produced by other organisms in the phylum Bacteroidetes. Like *B. fragilis*, the oral pathogen *Tannerella forsythia* produces an abundance of TBDTs and lipoproteins [79], but while this organism and the related oral pathogen *Porphyromonas gingivalis* secrete a variety of virulence factors, they use a pathway found exclusively in Bacteroidetes (the type IX pathway) instead of co-opting the pathways associated with Proteobacteria.

In addition to providing insight into the biology of a poorly characterized family of commensal bacteria that are major constituents of the human gut microbiome, our analysis of the secretion mechanisms used by *B. fragilis* has the potential to lead to the development of novel strategies to express and deliver beneficial proteins. Heterologous polypeptides fused to the C-terminal β barrel domain of *E. coli* and *Salmonella* autotransporters are often presented on the cell surface efficiently, and “autodisplay” has been used for the induction of immune protection and other biomedical applications [80]. Although the characterization of the type Vd pathway is still in its infancy, it is possible that polypeptides fused to the C terminus of the patatin domain-containing proteins produced by *B. fragilis* can be secreted in an analogous fashion. Cell surface display of proteins of interest might also be achieved by fusing them to localization signals contained within extracellular lipoproteins. Finally, insights gained from the identification and characterization of proteins that are substrates of the putative type I secretion systems might ultimately lead to a method to use *B. fragilis* to export heterologous polypeptides into the intestinal mlilieu.

## Supporting Information

S1 Text Supporting Materials and Methods. (DOCX)

S1 Table Proteins in OM fractions with ≥ 4 unique peptides identified by LC-MS/MS. (XLSX)

S2 Table Proteins in OM fractions with 2–3 unique peptides identified by LC-MS/MS. (XLSX)

S3 Table Peptides identified in abundant OM protein bands by mass spectrometry. (XLSX)

S4 Table Lipoproteins encoded adjacent to TBDT genes that are likely involved in nutrient uptake. (DOCX)

S5 Table Sulfo-NHS-SS-biotin-labeled proteins with ≥12 unique peptides identified by LC-MS/MS. (XLSX)

S6 Table Sulfo-NHS-SS-biotin-labeled proteins with 2–11 unique peptides identified by LC-MS/MS. (XLSX)

S7 Table Putative type I secretion and small molecule efflux pathway components produced in TYG. (DOCX)

S8 Table Putative type I secretion systems encoded in the *B. fragilis* genome. (DOCX)

S9 Table Proteins differentially expressed in different carbon sources. (XLSX)

S10 Table Proteins in the culture supernatant identified by LC-MS/MS. (XLSX)

S11 Table Peptides identified in abundant secreted protein bands by mass spectrometry. (XLSX)
