# Supplementary material for: Analysis of the Outer Membrane Proteome and Secretome of Bacteroides fragilis Reveals a Multiplicity of Secretion Mechanisms
Source: PLoS One. 2015 Feb 6;10(2):e0117732. doi: 10.1371/journal.pone.0117732 (PMC4319957; doi:10.1371/journal.pone.0117732)
Supplement: S1 Text — (DOCX) [file pone.0117732.s001.docx]

**SUPPORTING MATERIALS AND METHODS**

**General mass spectrometry methods**

All samples were analyzed using an inlet system comprised of a nanoscale UHPLC system (Waters NanoAcquity) operating with a vent valve, a 180 μm x 20 mm C18 trap column (Symmetry C18 5μm, Waters), and a 75 μm x 250 mm C18 analytical column (BEH130 C18 1.7μm, Waters). Both the trap column and the analytical column were maintained at 50^o^ C. The analytical column was connected to a fused silica emitter (New Objective FS360-20-10-CEL) through a conductive union (Upchurch M-572). The conductive union was held in a clip making electrical contact with a Proxeon nanospray source. The emitter was positioned approximately 5 mm from the orifice of the heated capillary of a Thermo Orbitrap Elite mass spectrometer along the axis of the capillary at an angle of ~15^o^ with the emitter tip lower than the union. The starting solution was 0.1% formic acid in water and the elution solution was 0.1% formic acid in acetonitrile.

Different types of LC-MS/MS experiments were performed to analyze complex protein mixtures and peptides eluted from gel slices. To analyze complex mixtures we used a longer gradient after a similar trapping phase. The gradient consisted of a 2 min threshold at 1% B, an increase from 1% B to 25% B in 538 min, a further jump to 80% B in 20 min and then a decrease to 1% B in 19 min. Up to 20 fragment spectra were collected after each parent scan. Due to a system volume related delay the mass spectrometer did not begin collecting data until 33 min into the gradient although FT measurements were made during this time to maintain the thermal equilibrium of the detector electronics. Once data collection began the mass spectrometer collected a single high-resolution mass spectrum (at the 240K setting) with a fill target of 1E6 which was followed by up to 20 lower resolution fragment mass spectra using a threshold of 5E3. Dynamic exclusion was used with two queries possible for typical peak widths, and charge state recognition prevented fragmentation of +1 and unknown charge states. Although data was collected for 460 min, essentially all of the detected peptides eluted in < 370 min. For the analysis of samples from in-gel digestions we used a shorter gradient comprised of a 10 min vented load followed by an increase from 1% B to 40% B in 20 min, a more rapid jump to 80% B, a 5 min plateau, and a 5 min return to the starting condition. During sample elution a top5 experiment was performed with dynamic exclusion with a count of 3 and a minimum threshold of 5000 with charge state recognition excluding +1 and unknown charge states. Parent spectra were collected with the 120K setting but preview was used to improve sampling.

MaxQuant 1.2 was used for the initial analysis of the OM proteome, and MaxQuant 1.3 was used for the analysis of all other complex protein mixtures [1]. Default settings were used to search a database of the hypothetical protein sequences of *B. fragilis* NCTC 9343 and common laboratory contaminants. When necessary, additional searches were performed using semi-trypsin cleavage. Mascot [2] was used to compare data obtained from gel slices against the same database.

Samples from in-gel digestion experiments were analyzed only using Mascot searches. In other experiments Mascot searches were performed in addition to MaxQuant searches in order to use earlier versions of Scaffold and to provide compatible input. For Mascot searches data were converted from Thermo.raw format to .mgf format using the Mascot Daemon, which in turn used the Thermo extractmsn program with default settings. Searches of the peak lists were then carried out using the Mascot Daemon and the NIH Center for Information Technology Mascot Cluster against a database of *B. fragilis* proteins (including hypothetical proteins) downloaded from UniProt in November 2012. A parent mass tolerance of 0.01 da (0.05 da for gel samples) and a fragment mass tolerance of 0.2 da were used, and one missed trypsin cleavage was allowed. Fixed modification with carbamidomethyl moieties and variable oxidation of methionine residues were also specified. MaxQuant searches generally included the same mass tolerances (although an internal mass recalibration was subsequently performed as part of data processing) and up to two missed cleavages were allowed. In dimethyl labeling experiments, only one missed cleavage was allowed. Analysis of data obtained from biotinylation experiments allowed for the modification of both the N-terminus of proteins and internal lysine residues with the reduced and carbamidomethylated form of Sulfo-NHS-SS-Biotin. This modification (with the composition H_7_C_5_O_2_NS) was specified separately for lysine and N-terminal residues within the Andromeda configuration utility.

By default the Mascot software performs searches with a 95% probability threshold. Important conclusions based on Mascot searches were limited to cases in which at least three separate peptides were identified to ensure a much lower probability of error. By default MaxQuant performs searches with a 1% false discovery rate based on a coincident search of artificial sequences. This approach leads to many protein identifications based on fewer than three peptides. At least three and often many more peptides were identified for individual proteins (or protein groups) discussed in the text, however. Although ratios were determined in experiments that involved PK treatment, it is important to distinguish these ratios from other types of ratios that we used. PK treatment often leads to the complete loss of certain peptides of a protein without affecting the levels of other peptides significantly. While the ratio of the total intensity for a protein can be thought of as an indication of the extent of its cell surface exposure, the ratio of the peptides detected typically has a wide distribution. Protein ratios in MaxQuant are not based on a mathematical averaging of observations, but instead involve the selection of a 50th percentile value. Because the ratios were of interest only when they were fairly large, non-normalized ratios were used and normalization of samples was based on the careful measurement of total protein concentrations.

**Preparation of SDS-PAGE bands**

Gel slices were destained and then incubated in an ion pairing extraction solvent of 85% acetone/5% water/5% acetic acid/5% triethylamine for 1 h on ice as described [3]. After extensive washing with 50% acetonitrile the samples were processed as described starting at the final dehydration step [4].

# Exploratory identification of OM proteins and initial comparison of samples from PK-treated and untreated cells

# For an exploratory identification of OM proteins and an initial comparison of OM proteins in PK-treated and untreated cells, samples were subjected to FASP [5] followed by a long gradient LC-MS/MS experiment. LFQ in MaxQuant [6] was used to compare raw signal intensities in separate data sets obtained from PK-treated and untreated cells.

# Quantitative comparison of protein samples using stable isotope dimethyl labeling

# Stable isotope dimethyl labeling was used to compare levels of individual proteins in PK-treated and untreated samples, cells grown in different media, and the culture medium versus the whole cell extract. To compare PK-treated and untreated samples and cells grown in different media, acetone-precipitated proteins were dissolved in 100 μl freshly prepared 100 mM Tris, pH 8/8M urea. Samples were incubated at 37^o^ C for 30 min with 10 μl 100mM DTT in 100mM Tris, pH 8/8M urea and at room temperature for 1 h with 15 μl 200 mM chloroacetamide in the dark. The alkylating agent was then scavenged by incubating samples at room temperature for 30 min with 15 μl 200 mM mercaptoethanol. Samples were incubated at room temperature overnight with Lys-C, which was added at a mass ratio of ~50:1. The samples were next diluted with three parts 100 mM NH_4_OAc and incubated at room temperature for a further 4 h with trypsin, which was added at a mass ratio of ~25:1. For the comparison of proteins in the culture medium versus the whole cell extract, care was taken to produce two samples that represented an equivalent number of cells. The samples were digested with Lys-C and trypsin as described in the original FASP protocol (5) and processed using the FASP method. In all experiments, individual samples were applied to a C18 Empore packed Stage Tip [7] and the bound peptides were then reductively methylated essentially as described (with proportional change in scale) using the 5C option [8]. Samples were then combined and analyzed using the long LC-MS/MS gradient described above.

**Sample preparation for identification of biotinylated proteins**

To retain equivalence with the use of SDS-PAGE during optimization, samples were eluted in SDS-PAGE sample buffer and subjected to the FASP protocol [5] using the reduction, alkylation, and serial Lys-C-trypsin digestion conditions that were recently described [9]. To avoid losses, digests were not filtered through the membrane but were removed carefully, washing the cartridge with 2 x 200 μl 100 mM NH_4_OAc and acidifying the combined sample by adding formic acid to a concentration of 5%. The digests were next applied in a cascade to a C8 200 μl Stage Tip (Proxeon) topped with ~50 μl of POROS R1 (50 μm, Life Technologies) with the flow-through applied to a C18 200 μl Stage Tip (Proxeon) topped with ~50 μl of POROS R2 (50 μm, Life Technologies). Again using a cascade (with the flow-through of the C8/R1 applied to the C18/R2) the columns were washed with 1x 300 μl 1.6% formic acid, 2x 300 μl 1.6% formic acid, 100 mM NH_4_OAc and 1x 200 μl 0.4% formic acid. The stack was reversed and peptides from the two columns were eluted using 300 μl 0.4% formic acid/40% acetonitrile followed by 300 μl 0.4% formic acid/80% acetonitrile. The eluate was then applied to an SCX 200 μl Stage Tip (Proxeon) topped with 50 μl of Source S (50 μm, GE Healthcare) subjected to the same pre-equilibration as described for the conventional SCX Stage Tip [7]. After each sample entered the bed the column was washed with 2x 300 μl 0.4% acetonitrile and 2x 300 μl 0.4% formic acid/80% acetonitrile. Peptides were eluted with 200 μl 0.4% formic acid/ 20% acetonitrile/500mM NH_4_OAc and 200 μl 0.4% formic acid/40% acetonitrile/200 mM NH_4_OAc. The combined sample was dried down with the sample vial held at 45^o^ C using a stream of room temperature nitrogen gas until a sparing volume was present. Subsequently 200 μl 50% acetonitrile was added and the sample was dried down until only a very small liquid residue remained and the cycle was repeated. Finally, the sample was resuspended in 30 μl 1.6% formic acid.

For analysis, 10 μl of each sample was loaded onto the trap column at a flow rate of 10 μl/min with the vent open. The vent was closed to create a connection to the analytical column. The stream passed through the trap column and the analytical column at a flow rate of 0.15 μl/min. After closing the vent a 5 min interval at 99% A was followed by an increase to 90% A in 45 min, an increase to 65% A in 230 min, a decrease to 35% A in 80 min, a decrease to 25% A in 10 min, and a return to 99% A in 10 min. Data was collected as described above in general mass spectrometry methods. The raw data was processed using MaxQuant 1.3 with a fixed CAM-cysteine modification and a variable modification of lysines and protein N-termini with C_5_O_2_NH_7_S. This modification results from the reduction and alkylation of the covalently linked biotinylation reagent. A similar search was also performed using Mascot using the Scaffold tool (Proteome Software) for visualization.

**SUPPORTING REFERENCES**

1. Cox J, Mann M (2008) MaxQuant enables high peptide identification rates, individualized p.p.b.-range mass accuracies and proteome-wide protein quantification. Nat Biotechnol 26: 1367-1372.

2. Perkins DN, Pappin DJ, Creasy DM, Cottrell JS (1999) Probability-based protein identification by searching sequence databases using mass spectrometry data. Electrophoresis 20: 3551-3567.

3. Zischka H, Gloeckner CJ, Klein C, Willmann S, Swiatek-de Lange M, et al. (2004) Improved mass spectrometric identification of gel-separated hydrophobic membrane proteins after sodium dodecyl sulfate removal by ion-pair extraction. Proteomics 4: 3776-3782.

4. Shevchenko A, Wilm M, Vorm O, Mann M (1996) Mass spectrometric sequencing of proteins silver-stained polyacrylamide gels. Anal Chem 68: 850-858.

5. Wiśniewski JR, Zougman A, Nagaraj N, Mann M (2009) Universal sample preparation method for proteome analysis. Nat Methods 6: 359-362.

6. Cox J, Hein MY, Luber CA, Paron I, Nagaraj N, et al. (2014) Accurate proteome-wide label-free quantification by delayed normalization and maximal peptide ratio extraction, termed MaxLFQ. Mol Cell Proteomics 13: 2513-2526.

7. Rappsilber J, Mann M, Ishihama Y (2007) Protocol for micro-purification, enrichment, pre-fractionation and storage of peptides for proteomics using StageTips. Nat Protoc 2: 1896-1906.

8. Boersema PJ, Raijmakers R, Lemeer S, Mohammed S, Heck AJ (2009) Multiplex peptide stable isotope dimethyl labeling for quantitative proteomics. Nat Protoc 4: 484-494.

9. Glatter T, Ludwig C, Ahrné E, Aebersold R, Heck AJ, et al. (2012) Large-scale quantitative assessment of different in-solution protein digestion protocols reveals superior cleavage efficiency of tandem Lys-C/trypsin proteolysis over trypsin digestion. J Proteome Res 11: 5145-5156.
