# Supplementary material for: Analysis of the Outer Membrane Proteome and Secretome of Bacteroides fragilis Reveals a Multiplicity of Secretion Mechanisms
Source: PLoS One. 2015 Feb 6;10(2):e0117732. doi: 10.1371/journal.pone.0117732 (PMC4319957; doi:10.1371/journal.pone.0117732)
Supplement: S4 Table — (DOCX) [file pone.0117732.s005.docx]

**Table S4. Lipoproteins encoded adjacent to TBDT genes that are likely involved in nutrient uptake**

| Locus tag | # Amino Acids | PK sensitive | Putative function | Predicted nearby or adjacent TBDT(s) |
| --- | --- | --- | --- | --- |
| BF9343_0985 | 214 | Yes | HmuY-like | BF9343_0986 |
| BF9343_1249 | 380 | Yes | Unknown | BF9343_1246 |
| BF9343_1250 | 337 | No | Unknown | BF9343_1246 |
| BF9343_1504 | 455 | Yes | Unknown | BF9343_1506 |
| BF9343_1505 | 680 | Yes | Unknown | BF9343_1506 |
| BF9343_1961 | 631 | No | Unknown | BF9343_1960 |
| BF9343_2074 | 472 | Yes | Unknown | BF9343_2073 and BF9343_2077 |
| BF9343_2076 | 215 | NF | Unknown | BF9343_2073 and BF9343_2077 |
| BF9343_2621 | 283 | Yes | Unknown | BF9343_2623 |
| BF9343_2979 | 194 | No | Unknown | BF9343_2982 |
| BF9343_2981 | 404 | Yes | Unknown | BF9343_2982 |
| BF9343_3058 | 532 | No | SusE homolog | BF9343_3060 |
| BF9343_3091 | 622 | Yes | Levanase | BF9343_3092 |
| BF9343_3115 | 626 | No | α-L-fucosidase | BF9343_3113 |
| BF9343_3171 | 328 | Yes | Unknown | BF9343_3170 |
| BF9343_3356 | 400 | Yes | Unknown | BF9343_3354 |
| BF9343_4107 | 1002 | No | Unknown | BF9343_4108 |
| BF9343_4228 | 406 | Yes | Unknown | BF9343_4229 |
