# Supplementary material for: Analysis of the Outer Membrane Proteome and Secretome of Bacteroides fragilis Reveals a Multiplicity of Secretion Mechanisms
Source: PLoS One. 2015 Feb 6;10(2):e0117732. doi: 10.1371/journal.pone.0117732 (PMC4319957; doi:10.1371/journal.pone.0117732)
Supplement: S7 Table — (DOCX) [file pone.0117732.s008.docx]

**Table S7. Putative type I secretion and small molecule efflux pathway components produced in TYG**

| Locus tag | # Amino acids | Predicted component type | Adjacent IM proteins |
| --- | --- | --- | --- |
| BF9343_0483 | 443 | TolC-like protein | ABC transporter |
| BF9343_0484 | 366 | Membrane fusion protein (IM protein) | ABC transporter |
| BF9343_2447 | 487 | TolC-like protein | ABC transporter |
| BF9343_2448 | 332 | Membrane fusion protein (IM protein) | ABC transporter |
| BF9343_2571 | 418 | TolC-like protein | ABC transporter |
| BF9343_0153 | 463 | Membrane fusion protein (lipoprotein) | AcrB family IM transporter |
| BF9343_0155 | 384 | Membrane fusion protein (lipoprotein) | AcrB family IM transporter |
| BF9343_0578 | 441 | TolC-like protein | AcrB family IM transporter |
| BF9343_1375 | 419 | TolC-like protein | None |
| BF9343_2394 | 468 | TolC-like protein | AcrB family IM transporter |
| BF9343_2395 | 379 | Membrane fusion protein (lipoprotein) | AcrB family IM transporter |
| BF9343_2859 | 458 | Membrane fusion protein (lipoprotein) | AcrB family IM transporter |
| BF9343_3064 | 353 | Membrane fusion protein (lipoprotein) | AcrB family IM transporter |
| BF9343_3066 | 424 | TolC-like protein | AcrB family IM transporter |
| BF9343_3105 | 459 | TolC-like protein | AcrB family IM transporter |
| BF9343_3107 | 355 | Membrane fusion protein (lipoprotein) | AcrB family IM transporter |
| BF9343_3276 | 548 | Membrane fusion protein (lipoprotein) | AcrB family IM transporter |
| BF9343_3277 | 501 | TolC-like protein | AcrB family IM transporter |
| BF9343_3420 | 392 | TolC-like protein | AcrB family IM transporter |
| BF9343_3858 | 338 | Membrane fusion protein (lipoprotein) | AcrB family IM transporter |
| BF9343_3859 | 439 | TolC-like protein | AcrB family IM transporter |
| BF9343_4200 | 430 | TolC-like protein | AcrB family IM transporter |
