# Supplementary material for: Analysis of the Outer Membrane Proteome and Secretome of Bacteroides fragilis Reveals a Multiplicity of Secretion Mechanisms
Source: PLoS One. 2015 Feb 6;10(2):e0117732. doi: 10.1371/journal.pone.0117732 (PMC4319957; doi:10.1371/journal.pone.0117732)
Supplement: S8 Table — (DOCX) [file pone.0117732.s009.docx]

**Table S8. Putative type I secretion systems encoded in the *B. fragilis* genome**

| Locus tags  (BF9343_) | TolC homolog  (Expressed protein with highest homology/E value) | MFP homolog  (Expressed protein with highest homology/E value) | ABC transporter  ATP binding protein homolog | ABC transporter  permease homolog |
| --- | --- | --- | --- | --- |
| 1850-1853 | 1853  (0483/4e^-46^) | 1852  (0484/5e^-29^) | 1851 | 1850 |
| 1527-1531 | 1527  (0483/2e^-22^) | 1528  (0484/2e^-8^) | 1530 | 1529, 1531 |
| 2370-2378 | 2370  (2571/0.31) | 2371  (2448/0.84) | 2378 | 2372-2377 |
| 3658-3661 | 3658  (0483/1e^-10^) | 3659  (2448/2e^-90^) | None | 3660, 3661 |

**TolC and MPF homologs were identified through BLAST searches in which the sequences of proteins that were produced in TYG were used to query the database of all proteins predicted to be produced by *B. fragilis* NCTC9343 (12). The E values obtained in the BLAST searches are shown. The presence of adjacent genes encoding ABC transporters suggested that the homologs are components of type I secretion systems. For simplicity, only the numerical portion of the locus tag (i.e., the four numbers following BF9343_) is shown.**
